# Thermosonication as a Novel Processing Technique to Enhance Phenolic Content, Amino Acids, and Health-Promoting Activities of White Onion Juice

**DOI:** 10.1021/acsomega.5c03006

**Published:** 2025-06-03

**Authors:** Dilek Dülger Altiner, Seydi Yıkmış, Esra Bozgeyi̇k, Melikenur Türkol, Filiz Aksu, Sema Sandıkçı Altunatmaz, Deniz Aktaran Bala, Selim Öğüt

**Affiliations:** † Department of Gastronomy and Culinary Arts, Tourism Faculty, 52980 Kocaeli University, Kartepe, Kocaeli 41400, Turkey; ‡ Department of Food Technology, Tekirdag Namik Kemal University, Tekirdag 59830, Turkey; § Department of Medical Biology, Faculty of Medicine, 162296 Adiyaman University, Adiyaman 02200, Turkey; ∥ Department of Nutrition and Dietetics, Faculty of Health Sciences, Tekirdag Namık Kemal University, Tekirdag 59030, Turkey; ⊥ Department of Food Processing, Vocational School of Veterinary Medicine, Istanbul University-Cerrahpaşa, Avcilar, Istanbul 34320, Turkey; # Department of Biophysics, Faculty of Medicine, Bandırma Onyedi Eylul University, Bandırma, Balıkesir 10250, Turkey

## Abstract

Onion (Allium cepa L.) possesses strong antioxidant, antimicrobial, and anti-inflammatory properties due to its rich content of flavonoids, phenolic compounds, vitamins, and minerals. This study aimed to evaluate the impact of thermosonication (TS) on enhancing the functional and bioactive properties of white onion juice. Three different treatments were applied: control (C-WOJ), thermal pasteurization (P-WOJ), and thermosonication (TS-WOJ). Using response surface methodology (RSM), the optimal TS conditions were determined as 45.45 °C, 12.90 min, and 67.67% amplitude. Under these conditions, total phenolic content (154.36 mg GAE/100 mL), total flavonoid content (19.62 mg CE/100 mL), and antioxidant activity (64.42%) significantly increased. TS also enhanced the levels of chlorogenic acid (355.71 μg/mL), catechin hydrate (123.86 μg/mL), and quercetin (153.13 μg/mL). Additionally, the TS group exhibited higher total amino acid content (15.64 mg/100 g), with notable increases in aspartic acid (3.82 mg/100 g) and glutamic acid (2.56 mg/100 g). Thermosonicated white onion juice demonstrated significant anticancer (inducing 11.9% apoptotic cell death in colon cancer cells), antihypertensive (ACE inhibition), and antidiabetic (α-glucosidase and α-amylase inhibition) effects. TS enhances the stability and bioavailability of bioactive components, offering a low-cost and effective alternative technology for the food industry.

## Graphical abstract

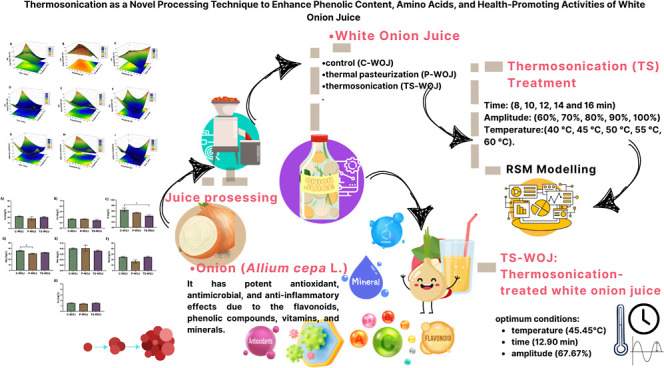

## 1 Introduction

Vegetables are crucial for well-being and nutrition because of their vitamins, minerals, phytochemicals, secondary compounds, and antioxidative substances. Onion (Allium cepa L.) is among the most widely consumed and produced vegetables globally, serving as a significant spice due to its distinctive aroma and flavor.^1^ Onion is a perennial species of the Amaryllidaceae family and Allium genus, characterized by aromatic subterranean stems.^2^ In the studies conducted, when the chemical structure of onion was examined, phenolic acids, flavonoids, anthocyanins, dipropyl disulfide, pyruvic acid, ascorbic, citric, malic acids, vitamin-C, Ca, P, K were detected, an average of 14% carbohydrates, 3–4% total sugar was reported.^3–6^ Onions have antioxidant, anti-inflammatory properties and support healthy bifidobacteria in the colon with the help of fructo-oligosaccharides. Quercetin (300 mg/kg) is 5–10 times higher in onions than in broccoli, blueberries and apples.^7^ The total phenolic content in white onion was reported as 7515 mg/g and antioxidant activity as 49.6%.^8^ Onion regulates metabolism and prevents diseases with the nutrients it contains. Onion is red, white, yellow, and green and can be consumed raw, cooked, roasted, dried, in salads, pickles, or in soup^6^

To ensure the safety and nutritional integrity of food, alternative technologies such as pasteurization have been recently developed. Especially in the food industry, products such as processed beverages that have undergone minimal processing and do not contain additives arouse interest.^9^ The thermosonication (TS) process, which has replaced thermal processing, is a new technology that uses ultrasound at higher temperatures and pressures. It combines heat and ultrasound and is used in the food industry to inactivate pathogens, spoilage organisms and enzymes, without altering food nutrients.^10^ The TS process improves juice quality/nutrition, enriches bioactive components and extends shelf life via changes to microbiology/functionality. Combining ultrasound and heat inactivates enzymes, stabilizing fruit/vegetable juices. The TS process preserves food with low energy/short processing time.^11^ Many researchers have tried the TS process, which is the application of ultrasound and heat, in different beverages such as carrot juice,^12^ blackberry juice,^13^ Berberis vulgaris juice,^14^ purple onion juice,^15^ prickly pear juice^16^ and investigated the effects of this alternative new technology on antioxidant, microbiological, nutritional and sensory parameters.

Optimization is the process of combining and using independent variables in a way that meets the defined goals, taking into consideration the interplay between the variables and how they impact the desired outcome. The response surface method (RSM) is a statistical optimization method that utilizes experimental strategies and modeling approaches to explain the relationship between a system’s response and independent variables, along with optimization methods to identify the levels at which the factors exert the desired influence on the system’s response.^17^

Through analysis of the examinations, it was observed that the bioavailability of white onion juice (WOJ) was elevated, and there was no application of ultrasound to maintain the stability of white onion juice. Ultrasound combined with moderate heat was more effective than ultrasound alone, so the aim was to produce high-quality white onion juice using RSM and optimization. The literature review found no studies on optimizing bioactive chemicals in white onion juice using RSM. This study aimed to examine the impact of pasteurization and thermosonication on the levels of bioactive compounds, phenolic compounds, antioxidant activity, minerals, fatty acid composition, free amino acids, organic acids, and sugar components in white onion juices (C-WOJ: untreated white onion juice, P-WOJ: thermal pasteurized white onion juice, and TS-WOJ: thermosonicated white onion juice). Additionally, the impact of this technology on white onion juice’s antidiabetic (α-glucosidase and α-amylase enzyme activity), antihypertensive (ACE-inhibitor), polyphenol oxidase enzyme (PPO) activity and anticancer (cytotoxicity, cell migration, colony formation, and cell death in colon (HT-29), breast (MCF-7) and lung (A549) cancer cells) activity was thoroughly investigated.

## 2 Materials and Methods

### 2.1 Processing of White Onion Juice

A white onion vegetable cultivated in the Thrace region served as the study’s essential material. White onions (A. cepa L.) used in the study were harvested in the Thrace region during the typical local harvest season, which spans from late June to early July 2023. The white onions are sorted from those that are not damaged or rotten. The washing process was thereafter executed. The white onions were peeled, and their outer skins were removed. White onions were ground in a Waring Industrial Mixer (model 8011ES, USA) at a low speed for 60 s, making pulp. The pulp was filtered. Citric acid (≥99.5% purity, Merck, Germany) was added to the prepared white onion juice (the pH value of the onion juice was approximately 4.3), and the acid adaptation process was carried out gradually. The prepared concentrates were stored in 100 mL sterile sample containers with lids at −18 °C. The study coded untreated white onion juice as C-WOJ. The samples underwent sensory screening, and the trained panel reported no off-flavors or unpleasant odors.

### 2.2 Thermal Pasteurization and Thermosonication Treatments

Thermal pasteurized white onion juice (P-WOJ) was generated by subjecting bottled white onion juice to pasteurization in a bath of water (Wisd-Model WUC-D06H, Daihan, Wonju, Korea) at 85 ± 1 °C for 10 min, followed by cooling to 20 ± 1 °C. An ultrasonic processor (Hielscher Ultrasonics, model UP200 St, Berlin, Germany) with a power output of 200 W was employed to treat 100 mL of white onion juice at a frequency of 26 kHz. Ultrasound was administered to the produced white onion juice at various temperatures (40, 45, 50, 55, and 60 °C), amplitudes (60, 70%, 80%, 90%, and 100%), and durations (8, 10, 12, 14, and 16 min). White onion juice that has been thermosonically treated is known as TS-WOJ. Throughout the procedure, the temperature was controlled with an ice block. Prior to analysis, samples were kept at −18 ± 1 °C.

### 2.3 Response Surface Methodology (RSM) Modeling Procedure

The influence of thermosonication treatment on the quality parameters of white onion juice was analyzed using Minitab Statistical Analysis Software (Minitab 18.1.1) and response surface methodology (RSM). The experimental design employed a five-level, three-factor configuration known as a central composite design (CCD). Twenty experimental points have been selected for optimization (Table 1). The model’s adequacy was evaluated using *R* ^2^ and adjusted *R* ^2^ coefficients, lack-of-fit examinations, and ANOVA results. The ultrasonic frequency was determined at 26 kHz, with the independent variables identified as temperature (*X*_1_), time (*X*_2_), and amplitude (*X*_3_). The dependent variables included ascorbic acid, total phenolic content, flavonoid content, and antioxidant activity. The model equations were formulated with a second-degree polynomial equation, as illustrated in the formula following.

1 $$y=\beta_{0}+\sum_{i=1}^{3}\beta_{i}X_{i}+\sum_{i=1}^{3}\beta_{ii}X_{i}^{2}+\sum_{\begin{array}{l} i=1\\ i<j \end{array}}^{3}\;\sum_{j=1}^{3}\beta_{ij}X_{i}X_{j}$$

**Table 1:** Coded and Uncoded Values of Independent Variables

	factor levels				
independent variables	–1.68	–1	0	1	1.68
*X*_1_: temperature (°C)	40	45	50	55	60
*X*_2_: time (min.)	4	7	10	13	16
*X*_3_: amplitude (%)	60	70	80	90	100

The subsequent formula is followed: *X*_i_ and *X*_*j*_ represent the independent variables, β_*i*_ denotes the coefficient of the first-order (linear) term, β_*ii*_ signifies the coefficient of the quadratic term, and β_*ij*_ indicates the coefficient of the two-factor interaction term.

### 2.4 Determination of Bioactive Compounds

The Folin-Ciocalteau methodology^18^ was applied to quantify the total phenolic content (TPC). The total flavonoid concentration (TFC) was determined using the colorimetric method with aluminum chloride.^19^ The results indicated the total flavonoid concentration in milligrams of catechin equivalents (CE) per liter. The DPPH (2,2-diphenyl-1-picrylhydrazyl) method was applied to evaluate DPPH activity with the further changes.^20^ The absorbance was subsequently quantified at 517 nm utilizing a UV–vis spectrophotometer (SP-UV/vis-300SRB, Spectrum Instruments, Melbourne, Australia). The DPPH scavenging activity was determined using eq 2.

2 $$DPPH\;radical\;scavenging\;activity\left(\%\right)=\left(A_{0}-A_{1}\right)/A_{0}\times 100$$

*A*_0_ indicates the absorbance of the control, while *A*_1_ signifies the absorbance of the white onion juice. The CUPRAC (cupric reducing antioxidant capacity) method was employed to assess the capacity of white onion juice to reduce copper ions, hence determining its overall antioxidant activity.^21^ The findings were presented as a percentage of inhibition. Every sample was examined three times.

### 2.5 Determination of Antihypertensive (ACE-Inhibitor) Activity

Modifications were made to the ACE inhibitory activity assay.^22^ The technique uses hippuric acid as a measure. A sodium borate buffer with NaCl was used to dissolve the acid and make the solution. The buffer was also used for the enzyme. Twenty μL of the HHL was added to the protein hydrolysate, which was then left for 5 min at 37 °C. Then 20 μL of ACE was added and the mixture was left again for 30 min 100 μL of HCl was added to stop the reaction, and 1.2 mL of ethyl acetate was added to isolate the acid. The material was vortexed and spun at 8000 rpm for 10 min. To ensure the complete evaporation of ethyl acetate, 1 mL of the upper ethyl acetate phase was meticulously transferred to a clean test tube and heated to 95 °C for 20 min following centrifugation. Following the dissolution of the residual substance in 3 mL of distilled water, the absorbance at 228 nm was recorded. The subsequent formula was employed to ascertain the ACE inhibitory activity

3 ACEinhibitoractivity=[(A−B/A−C)]×100

*A* = ACE + HHL absorbance, *B*= Sample + HHL absorbance, *C* = ACE + HHL + Sample absorbance.

### 2.6 Determination of Antidiabetic Activity

The α-glucosidase and α-amylase enzyme activity were assessed using a modified process to evaluate the potential of hawthorn vinegar as an antidiabetic agent.^23^ Acarbose served as the positive control in these analyses. A UV–vis spectrophotometer (Spectrum Instrument, SP-UV/vis-300SRB, Australia) was applied for measuring absorbance.

### 2.7 Measurement of Polyphenol Oxidase Enzyme (PPO) Activity

The assay for PPO activity followed the Ndiaye et al. (2009)^24^ method. Catechol (purity ≥99%, Sigma-Aldrich, USA) solution (0.05 mol/L, diluted in 0.2 M phosphoric acid, pH 6.5) and 0.1 mL of crude enzyme extract were combined. The reaction was measured at 420 nm for 3 min. A 3 mL catechol solution without enzyme extract was also tested as a blank. The assay was performed 3 times. The following formula was used to determine PPO’s relative residual activity (RA)

4 $$RA=\frac{A_{t}}{AA_{0}}\times 100\%$$

where *A*_0_ represents the initial PPO activity before treatment, and *A*_*t*_ denotes the residual PPO activity at a specific time *t*. The enzyme activity was measured as the quantity of enzyme that induced an absorbance variation of 0.001 per minute under the established testing conditions.

### 2.8 Phenolic Compounds (HPLC)

Chromatography was conducted by Portu et al. (2017)^25^ utilizing a C-18 (250 × 4.6 mm; 5 μm packing; Agilent) ACE Genix column. The study was conducted using an Agilent HPLC system (model 1260 Infinity LC, Agilent Technologies, Santa Clara, CA, USA). A DAD-equipped Agilent 1260 chromatograph was used to study the polyphenols. A flow rate of 0.80 mL/min was used. The column temperature was set at 30 °C. Gradient elution was performed using eluents A and B. Acetonitrile constituted solution B, while water with 0.1% phosphoric acid constituted solution A. The gradients used were 17% at 0 min, 15% at 7 min, 20% at 20 min, 24% at 25 min, 30% at 28 min, 40% at 30 min, 50% at 32 min, 70% at 36 min and 17% at 40 min. Standard solutions of each drug were injected to ascertain their retention time. The injection volume for phenolic analysis was 10 μL. The UV–vis spectrophotometer was used for analysis at wavelengths of 280, 320, and 360 nm, with values expressed as μg/mL sample. Standard solutions of these sugars were also injected to confirm their retention time.

### 2.9 Components of Organic Acids and Sugars (HPLC)

The organic acid content was assessed with high-performance liquid chromatography (HPLC) with minor changes to the methodology established by Coelho et al. (2018).^26^ The study was conducted using an Agilent HPLC system to separate and detect the organic acids. A 20 μL aliquot was injected into the system after filtration through a 0.45 μm syringe filter. An Agilent Hi-Plex H ion-exchange column (300 × 7.7 mm) was used. Results were expressed as grams per liter (g/L) of substance. A RID was used to identify fructose, sucrose and glucose. The acids and sugars were quantified using the method described by Coelho et al. (2018), with results in g/L.

### 2.10 Determination of Free Amino Acid (LC–MS)

A modified version of the methodology proposed by Bilgin et al. (2019)^27^ was employed to ascertain the amino acid composition. Chromatographic separation was achieved in 7.5 min using a flow rate of 0.7 mL/min and gradient-programmed mobile phases A and B. A triple quadrupole LC–MS system (Agilent 6460, Agilent Technologies, Waldbronn, Germany) equipped with an electrospray ionization interface was utilized for the MS/MS analysis. The settings set for the mass spectrometer are a capillary voltage of +2000 V, a nebulizer pressure of 40 psi, a gas temperature of 150 °C, and a gas flow rate of 10 L/min. Each sample was analyzed thrice. The stated result is mg/100 mL.

### 2.11 Determination of Minerals (ICP-OES)

The mineral content of white onion juice was examined utilizing a methodology developed by Sezer et al. (2019).^28^ Analyses of calcium (Ca), copper (Cu), iron (Fe), manganese (Mn), magnesium (Mg), sodium (Na), potassium (K), and zinc (Zn) content in the samples were performed utilizing inductively coupled plasma optical emission spectrometry (ICP-OES) (PerkinElmer 2100 Dual View, USA). The detected analytical lines are Ca at 317.9 nm, Fe at 238.2 nm, Mn at 257.6 nm, Mg at 285.2 nm, P at 213.6 nm, Zn at 206.2 nm, Na at 589.5 nm, and K at 766.5 nm.

### 2.12 Anticancer Activity

To test anticancer activity of white onion juice, we assayed cytotoxicity, cell migration, colony formation, and cell death in colon (HT-29), breast (MCF-7) and lung (A549) cancer cells using MTT [3-(4,5-dimethylthiazol-2-yl)-2,5-diphenyltetrazolium bromide] (purity ≥98%, Merck, Germany) cell viability assay, wound healing assay, colony forming assay and Annexin V/PI double staining assay, respectively. HT-29, MCF-7, and A549 cancer cell lines were obtained from the American Type Culture Collection (ATCC). The cells were grown in RPMI-1640 (HyClone) enriched with 10% fetal bovine serum (HyClone) and 1% streptomycin/penicillin/fungizone antibiotic-antimycotic mixture (HyClone) at 37 °C in a 5% CO_2_ incubator. Upon achieving sufficient confluency, the cells were detached using Trypsin–EDTA (HyClone) and utilized for following investigations.

#### 2.12.1 Determination of Cytotoxicity Using the MTT Assay

The MTT cell viability experiment was performed as previously outlined in.^29^ The cells were seeded to 96-well plates at a density of 2 × 10̂5 cells/mL, with 100 μL dispensed per well, and cell density was assessed the same day. The cells were subsequently subjected to varying doses of WOJ (31.2, 62.5, 125, 250, and 500 μL/mL). Twenty-4 h after exposure, the cells were stained with 1 mg/mL MTT [3-(4,5-dimethylthiazol-2-yl)-2,5-diphenyltetrazolium bromide] (Merck, purity 98.0%) and incubated for 2 h. The growth media was discarded postincubation, and the formazan crystals were solubilized in Dimethyl sulfoxide (Merck, purity ≥99.0%). Absorbance was quantified at a wavelength of 550 nm utilizing a microplate reader (Thermo, Multiskan GO, Thermo Fisher Scientific). Cell viability was determined using optical density (OD) data.^30^

#### 2.12.2 Determination of Cell Migration by Wound Closure Assay

The cells were seeded to 6-well culture plates for the wound closure experiment and permitted to establish a monolayer. After establishing a monolayer, a wound (gap) was generated in the center of the plate utilizing a 100 μL pipet tip. The plates underwent two washes with 1X PBS (HyClone), and pictures were captured on day 0. Thereafter, the cells were subjected to 250 μL/mL WOJ samples, and photos of the wound gap were captured every 24 h to evaluate the cell migration capability in comparison to the control group. The wound gap measurements in the acquired pictures were conducted utilizing ImageJ software.

#### 2.12.3 Colony Formation Assay

In the colony formation assay, cells were plated to 12-well plates at a density of 1 × 10^3^ cells/mL. Subsequently, the cells were subjected to 50 μL/mL samples of WOJ. When each colony contained at least 50 cells, the medium was aspirated, and the cells were fixed with 70% ethanol (Isolab, purity ≥99.9%). Then, 0.5% crystal violet (Sigma-Aldrich; purity ≥90%) was added, and the cells were maintained in dark. Following incubation, well were rinsed with ddH_2_O until colonies made visible.

#### 2.12.4 Determination of Apoptosis by Annexin V/PI Double Staining

For the apoptosis experiments, the cells were plated into 6-well plates at a density of 5 × 10̂6 cells/mL. Cells treated with 250 μL/mL WOJ were utilized for apoptosis assays employing the Annexin V/PI kit (BD Pharmingen). The cells labeled with Annexin V/PI were assessed using a C6 Flow Cytometer to identify the populations of apoptotic, necrotic, and viable cells.

#### 2.12.5 DNA Fragmentation Assay

For the DNA fragmentation examination, the cells were seeded into 12-well plates at a density of 2 × 10^5^ cells/mL. On the subsequent day, the cells were subjected to 250 μL/mL samples of WOJ. Following a 24 h period, the cells were harvested, and DNA extraction was conducted with the GeneAll DNA Isolation Kit (GeneAll Biotechnology Co., Ltd.). Following isolation, the purity and concentration of DNA samples were assessed utilizing a spectrophotometer. The DNA was then subjected to electrophoresis on a 1% agarose gel and visualized using UV light.

### 2.13 Statistical Analysis

Statistical analyses for the study were performed utilizing SPSS 20.0 (SPSS Inc., Chicago, U.S.A) and GraphPad Prism software. Samples were evaluated using a one-way ANOVA accompanied by Tukey’s multiple comparison test. The threshold for statistical significance was set at p < 0.05. The response surface methodology was executed utilizing Minitab statistical analysis software (Minitab 18.1.1).

## 3 Result and Discussion

### 3.1 Optimization of Bioactive Compounds

RSM is a statistical approach that conserves time and effort in content optimization.^31^ RSM was employed to assess the impact of thermosonication on the total phenolic content (TPC), total flavonoid content (TFC), and antioxidant activity (DPPH) in white onion juice. In the thermosonication process, temperature (*X*_1_, 40–60 °C), time (*X*_2_, 4–16 min), and amplitude (*X*_3_, 40–60%) were designated as independent variables, while total phenolic content (TPC, mg GAE/100 mL), total flavonoid content (TFC, mg CE/100 mL), and DPPH (% inhibition) served as the response variables. A central composite design (CCD) response surface methodology was employed to optimize the thermosonication process for white onion juice, utilizing Minitab software (Minitab Software, State College, PA, USA, Version 19). The cumulative count of experiments from the three-factor CCD was 20 (Table 2), with each experiment replicated three times. Response Surface Methodology (RSM) was employed to maximize the bioactive constituents of white onion juice. Consequently, the optimization yielded eq 5 for the second-order modeling of the TPC (mg GAE/100 mL), eq 6 for the TFC (mg CE/100 mL), and eq 7 for the DPPH (% inhibition).

5 $$TPC(mgGAE/100mL=-110.8+2.966X_{1}+9.334X_{2}+3.470X_{3}-0.14027X_{1}X_{1}-0.30845X_{2}X_{2}-0.045120X_{3}X_{3}+0.1823X_{1}X_{2}+0.11033X_{1}X_{3}-0.14609X_{2}X_{3}$$

6 $$TFC\left(mgCE/100mL\right)=251.12-6.097X_{1}-2.582X_{2}-1.7717X_{3}+0.03213X_{1}X_{1}+0.05972X_{2}X_{2}+0.006629X_{3}X_{3}+0.10117X_{1}X_{2}+0.02340X_{1}X_{3}-0.04481X_{2}X_{3}$$

7 $$DPPH\left(\%\;inhibition\right)=743.0-19.491X_{1}-4.154X_{2}-4.574X_{3}+0.11356X_{1}X_{1}+0.2920X_{2}X_{2}+0.01635X_{3}X_{3}+0.2417X_{1}X_{2}+0.07254X_{1}X_{3}-0.17288X_{2}X_{3}$$

**Table 2:** Determined Responses Utilized in the Experimental Model for Response Surface Methodology (RSM) *X*_*1*_: Temperature; *X*_*2*_: Time; *X*_*3*_: Amplitude; RSM: Response Surface Methodology; TS-WOJ: Thermosonicated White Onion Juice; TPC: Total Phenolic Content; TFC: Total Flavonoid Content; DPPH: Antioxidant Activity (2,2-Diphenyl-1-picrylhydrazyl)

				dependent variables					
	independent variables			TPC (mg GAE/100 mL)		TFC (mg CE/100 mL)		DPPH (% inhibition)	
run no	temperature (*X*_1_)	time (*X*_2_)	amplitude (*X*_3_)	experimental data	RSM predicted	experimental data	RSM predicted	experimental data	RSM predicted
1	50	16	80	146.17	146.20	18.35	18.44	61.38	61.59
2	50	10	80	153.73	153.75	15.71	15.77	51.32	51.44
3	45	7	90	147.68	147.90	19.34	19.32	63.16	62.88
4	50	10	100	141.83	141.83	18.57	18.64	56.58	56.78
5	50	10	80	154.37	153.75	15.71	15.77	51.32	51.44
6	55	13	90	149.89	149.61	19.63	19.49	60.88	60.25
7	45	7	90	147.79	147.90	19.35	19.32	63.23	62.88
8	50	10	80	153.73	153.75	15.71	15.77	51.32	51.44
9	50	10	80	153.73	153.75	15.71	15.77	51.32	51.44
10	55	10	70	135.58	135.09	16.25	15.96	53.97	53.31
11	50	4	80	139.61	139.09	17.32	17.41	61.93	62.30
12	50	10	80	153.73	153.75	15.71	15.77	51.32	51.44
13	60	10	80	135.4	135.61	18.76	18.98	63.15	63.64
14	45	7	70	143.64	144.04	18.81	18.76	61.27	60.95
15	50	10	60	129.96	129.57	18.02	18.21	58.62	59.17
16	40	10	80	144.43	143.84	18.96	18.99	61.69	61.94
17	45	13	70	150.58	150.89	19.04	18.93	64.42	63.72
18	55	7	90	149.54	149.35	18.7	18.62	63.98	63.73
19	55	7	70	123.02	123.42	13.44	13.38	47.9	47.30
20	50	10	80	153.73	153.75	15.71	15.77	51.32	51.44
TS-WOJ (RSM optimization parameters)	45.45	12.90	67.67	154.36	19.62	64.42			
experimental values				149.70	18.58	62.13			
% difference				3.01	5.12	3.55			

Thermosonication is an effective technique for augmenting the bioactive constituents of white onion juice. The optimization led to studies examining the impacts of temperature, duration, and amplitude on total phenolic content (TPC), total flavonoid content (TFC), and antioxidant activity (DPPH). Under the optimal conditions determined by RSM (45.45 °C, 12.90 min, 67.67% amplitude), the anticipated values for TPC, TFC, and DPPH were 154.36 mg GAE/100 mL, 19.62 mg CE/100 mL, and 64.42%, respectively. The experimental values reported under identical conditions were 149.70 mg GAE/100 mL, 18.58 mg CE/100 mL, and 62.13%, respectively. These minor differences (%3.01%, 5.12%, and %3.55%) are a natural result of experimental variability and support the overall accuracy and reliability of the RSM model. This study is significant in demonstrating the potential of thermosonication for preserving and enhancing the functional components of white onion juice. The close alignment between experimental results and model predictions shows that RSM can be effectively used as an optimization tool in food processing. Other studies employing RSM have also consistently aligned experimental results and model predictions.^32–34^ ANOVA results of the white onion juice study are given in Table 3.

**Table 3:** ANOVA Findings of Regression Coefficients Derived From RSM of TPC, TFC, and DPPH Responses due to Thermosonication^a^

		TPC (mg GAE/100 mL)		TFC (mg CE/100 mL)		DPPH (% inhibition)	
source	DF	*F*-value	*P*-value	*F*-value	*P*-value	*F*-value	*P*-value
model	9	846.520	0.000	253.830	0.000	227.530	0.000
linear	3	285.480	0.000	12.570	0.001	6.360	0.011
*X*_*1*_	1	259.600	0.000	0.000	0.948	7.450	0.021
*X*_*2*_	1	213.720	0.000	33.050	0.000	1.430	0.259
*X*_*3*_	1	576.100	0.000	5.300	0.044	14.890	0.003
square	3	1229.150	0.000	309.040	0.000	362.380	0.000
*X*_*1*_ *X*_*1*_	1	1571.590	0.000	615.190	0.000	689.230	0.000
*X*_*2*_ *X*_*2*_	1	992.450	0.000	277.650	0.000	595.300	0.000
*X*_*3*_ *X*_*3*_	1	2601.580	0.000	419.140	0.000	228.460	0.000
2-way interaction	3	434.360	0.000	240.890	0.000	215.610	0.000
*X*_*1*_ *X*_*2*_	1	182.430	0.000	419.330	0.000	214.550	0.000
*X*_*1*_ *X*_*3*_	1	1070.920	0.000	359.500	0.000	309.730	0.000
*X*_*2*_ *X*_*3*_	1	468.600	0.000	329.110	0.000	439.110	0.000
error	10						
lack-of-fit	4	7.100	0.018	3714.440	0.000	1802.520	0.000
pure error	6						
Total	19						
*R* ^2^		99.87%	99.56%	99.51%			
adj *R* ^2^		99.75%	99.17%	99.08%			
pred. *R* ^2^		98.67%	96.56%	95.20%			

a *X*_1_: temperature; *X*_2_: time; *X*_3_: amplitude; DF: degrees of freedom; *R* ^2^: coefficient of determination; TPC: total phenolic content; TFC: total flavonoid content; DPPH: antioxidant activity (2,2-diphenyl-1-picrylhydrazyl); *p* < 0.05: statistically significant; *p* < 0.01: statistically very significant.

ANOVA analysis offers significant statistical validity in evaluating the impact of the thermosonication method on TPC, TFC, and antioxidant activity (DPPH) in white onion juice. The RSM model is statistically significant for all variables that responded (*p* < 0.0001). The model’s coefficient of determination (*R* ^2^) is above 99% for all responses (TPC: 99.87%, TFC: 99.56%, DPPH: 99.51%), indicating that the model represents the experimental data with high accuracy. All linear, quadratic, and interaction terms show statistically significant at the *p* < 0.05 level, demonstrating that temperature, time, and amplitude all impact the process. *X*_3_ (amplitude) is notably one of the most significant determinants of both phenolic content and antioxidant activity (*p* < 0.01). Figures A, B, and C demonstrate the variations in TPC values in relation to temperature, time, and amplitude. In Figure A, TPC increases with the rise in temperature and time, while Figure B shows an increase in phenolic content with the rise in amplitude. Figure C illustrates the effect of the combination of time and amplitude. However, the effect of *X*_2_ (time) on flavonoid content is more pronounced (*p* < 0.0001). Figures D, E, and F show the changes in TFC. Figure D indicates the impacts of temperature and duration, Figure E depicts the influence of amplitude on flavonoid concentration, and Figure F demonstrates the synergistic effects of time and amplitude. Figures G, H, and I illustrate antioxidant activity (DPPH). Figure G illustrates the impact of temperature and duration, Figure H depicts the influence of amplitude, and Figure I emphasizes the synergistic effects of amplitude and duration. The augmentation of processing time and amplitude positively influenced the TPC, TFC, and DPPH outcomes, matching with prior research.^15,35,36^

The in two directions interactions between *X*_1_–*X*_3_ and *X*_2_–*X*_3_ are markedly significant for all response variables (*p* < 0.0001), suggesting that temperature, amplitude, and time can significantly impact the stability of functional components. The quadratic effects (*X*_1_^2^, *X*_2_^2^, *X*_3_^2^) with the greatest F values for all variables indicate that the response variables demonstrate nonlinear behavior, confirming the value of the RSM model for process optimization. The predicted *R* ^2^ values of the model (TPC: 98.67%, TFC: 96.56%, DPPH: 95.20%) are also relatively high, supporting the overall predictive power of the model. These findings validate the potential of the thermosonication process in preserving and enhancing the bioactive components of white onion juice, highlighting the importance of optimization studies in food processing technologies. Similar results have been achieved in other studies.^37,38^ The improvement of bioactive components is due to the increased permeability of the cell wall resulting from the interplay of cavitation and temperature during the thermosonication process (see Figure 1).

**Figure 1:**
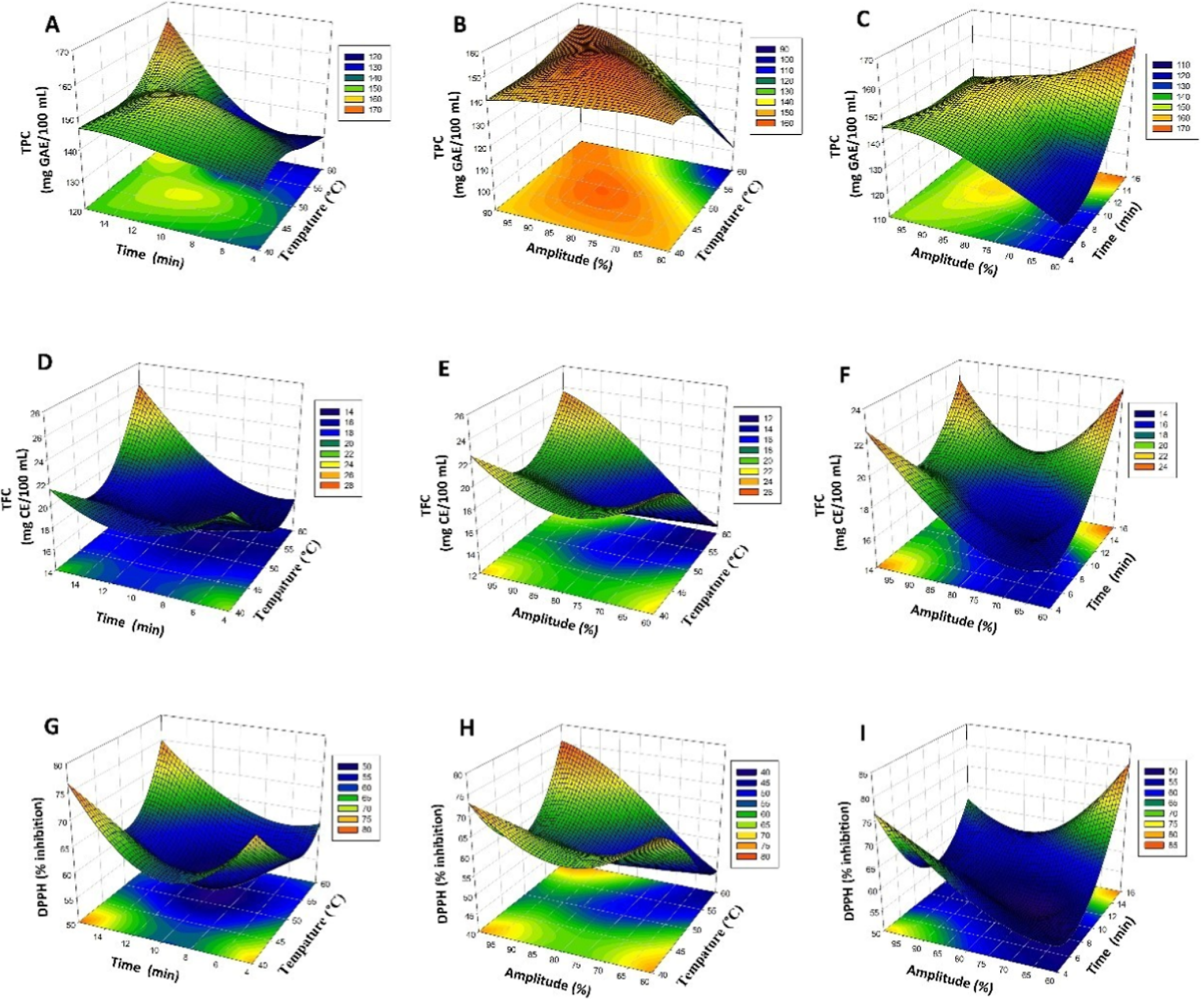
Three-dimensional (3D) mesh plots (A-I) illustrating the impact of thermosonication on total phenolic content (TPC), total flavonoid content (TFC), and antioxidant activity (DPPH) in white onion juice utilizing response surface methodology (RSM).

### 3.2 Bioactive Compounds

Onions show antioxidant and antimicrobial effects with the function of anthocyanins, flavonoids, organosulfur compounds, quercetin, kaempferol, and polyphenol compounds inside of onions.^6^ Onion contains pharmacological capabilities and exhibits hypolipidemic, anti-inflammatory, antihypertensive, antidiabetic and immunoprotective actions due to its bioactive components.^1^ The principal phenolic chemicals found in onions include ferulic acid, gallic acid, protocatechuic acid, and quercetin.^8^ Figure 2 shows the comprehensive results for total phenolic content (TPC), total flavonoid content (TFC), radical scavenging activity (DPPH), and cupric ion-reducing antioxidant capacity (CUPRAC) in white onion juice.

**Figure 2:**
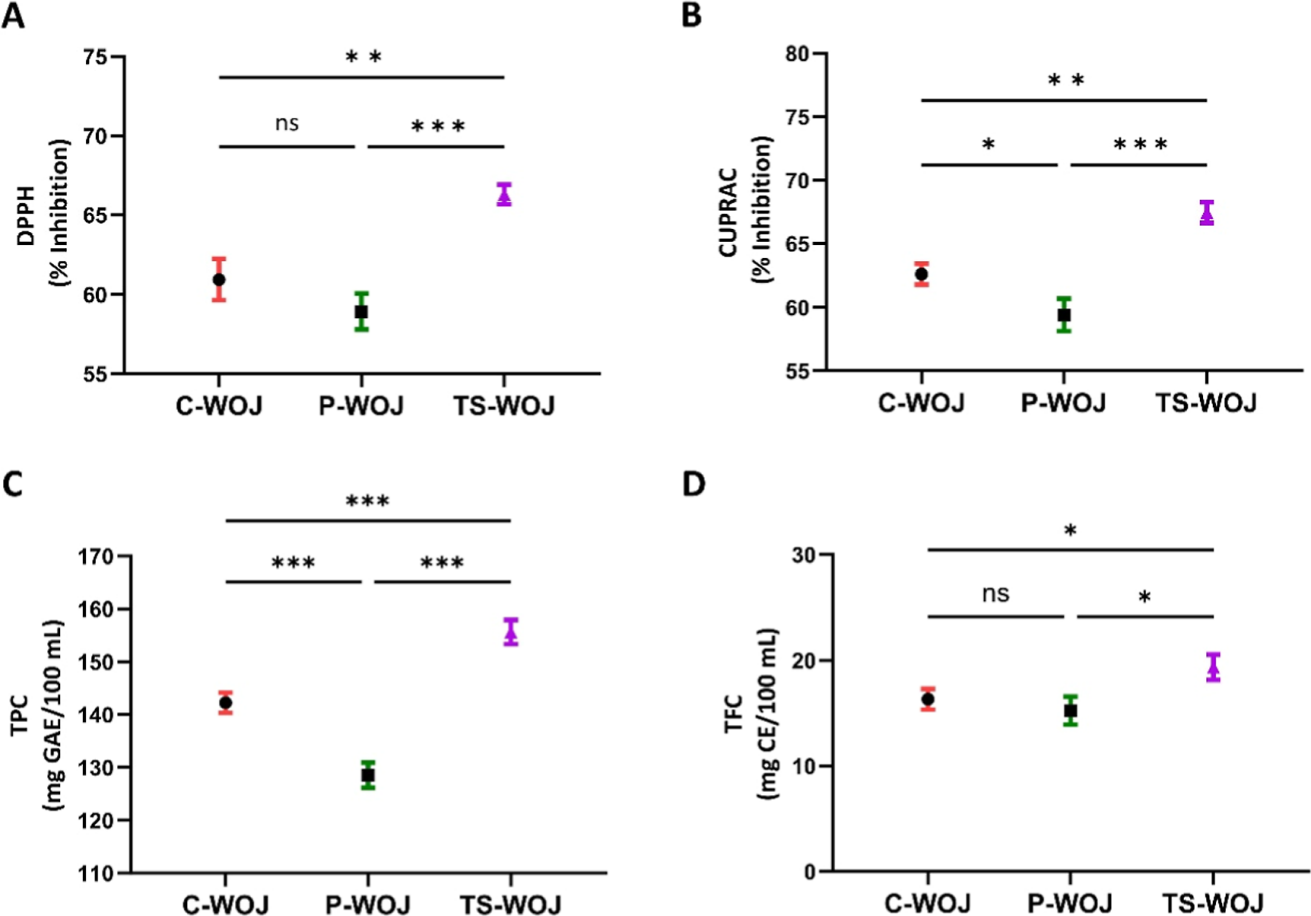
Radical scavenging activity (DPPH) (A), and cupric ion reducing antioxidant capacity (CUPRAC) (B), total phenolic content (C), total flavonoid content (D), of White onion juice. (ns: insignificant; *p < 0.05; **p < 0.01, ***p < 0.001, (*n* = 3 ± SD)). C-WOJ: control white onion juice; P-WOJ: thermal pasteurized white onion juice; TS-WOJ: thermosonication-treated white onion juice.

The inhibition percentages for DPPH and CUPRAC in the C-WOJ, P-WOJ, and TS-WOJ samples were 62.61% and 60.95%, 58.93% and 59.41%, and 66.30% and 67.45%, respectively (Figure A,B). No significant statistical difference was detected between the untreated control and thermal pasteurized material in the analysis of DPPH activity. The CUPRAC method demonstrated a statistically significant difference (*p* < 0.05) in the percentage inhibition values between C-WOJ and P-WOJ examples. In TS-WOJ samples, treated with two distinct antioxidant capacity analyses, the highest percentage inhibition values were observed (*p* < 0.01). Nonthermal technologies with little or no effect on food products are proposed to reduce pasteurization’s negative effects. The DPPH method is extensively utilized for its simplicity and effectiveness to evaluate the antioxidant capacity of plant products.^39^ Our investigation found that TS treatment positively influenced the antioxidant capacity of white onion juice. Based on the outcomes of bioactive substances, The TS treatment enhanced antioxidant activity of blackthorn vinegar,^40^ purple onion juice,^15^ and posotia (Vitex negundo) juice.^41^ The thermosonication technique is proposed as an alternative to thermal methods in fruit juice processing.^41^ Another study revealed that the onion juice utilized enhanced the antioxidant potential of fermented milk, as measured by DPPH method.^42^ Dulger Altıner et al. (2024) similarly reported a decrease in DPPH levels in pasteurized WOJ compared to C-WOJ.^43^

Thermosonication (15 and 25 min, 80% amplitude) applied to prickly pear juice increased phenolic content after 14 days of storage, particularly for the treatment at 80% amplitude for 25 min, along with an increase in antioxidant activity (ABTS, DPPH) throughout the storage period.^44^ Thermosonication refers to the integration of ultrasound with mild thermal treatments.^45^ The TS process is crucial for stability, enhancement of bioactive substances, improvement of sensory attributes, and augmentation of product quality and shelf life.^46^

The TPC results for C-WOJ, P-WOJ, and TS-WOJ samples were 142.27, 128.55, and 155.64 mg GAE/100 mL, respectively (Figure 4C). The total phenolic contents of the samples varied, with thermosonicated white onion juice presenting the highest TPC result (*p < 0.001). The results indicate that thermosonication is superior to heat pasteurization in enhancing total phenol content. Similar to the increase in TPC results of white onion juice, similar superiorities were reported in products such as thermosonication-treated (TS) parsley juice,^43^ and purple onion juice.^15^ Likewise, in a study conducted with posotia leaf (V. negundo) juice, when the TPC results were examined, the pasteurized sample (141.39 mg of GAE/100 mL) showed lower results than the raw juice sample (148.098 mg GAE/100 mL).^41^ A study similar to ours on cranberry juice shown that the maximum total phenolic, flavonoid, and antioxidant activity values were attained with thermosonication treatment at 65 °C-75 °C with 50% processed ultrasound amplitude for 30 min.0The TS process is thought to improve cell wall permeability by the cavitation and warmth it generates in the product, leading to an increase in bioactive chemicals. Numerous studies have indicated that phenolic compounds mitigate the risk of cardiovascular disease and obesity due to their antioxidant, anticancer, antifungal, antibacterial, and antidiabetic activities.^47^

**Figure 3:**
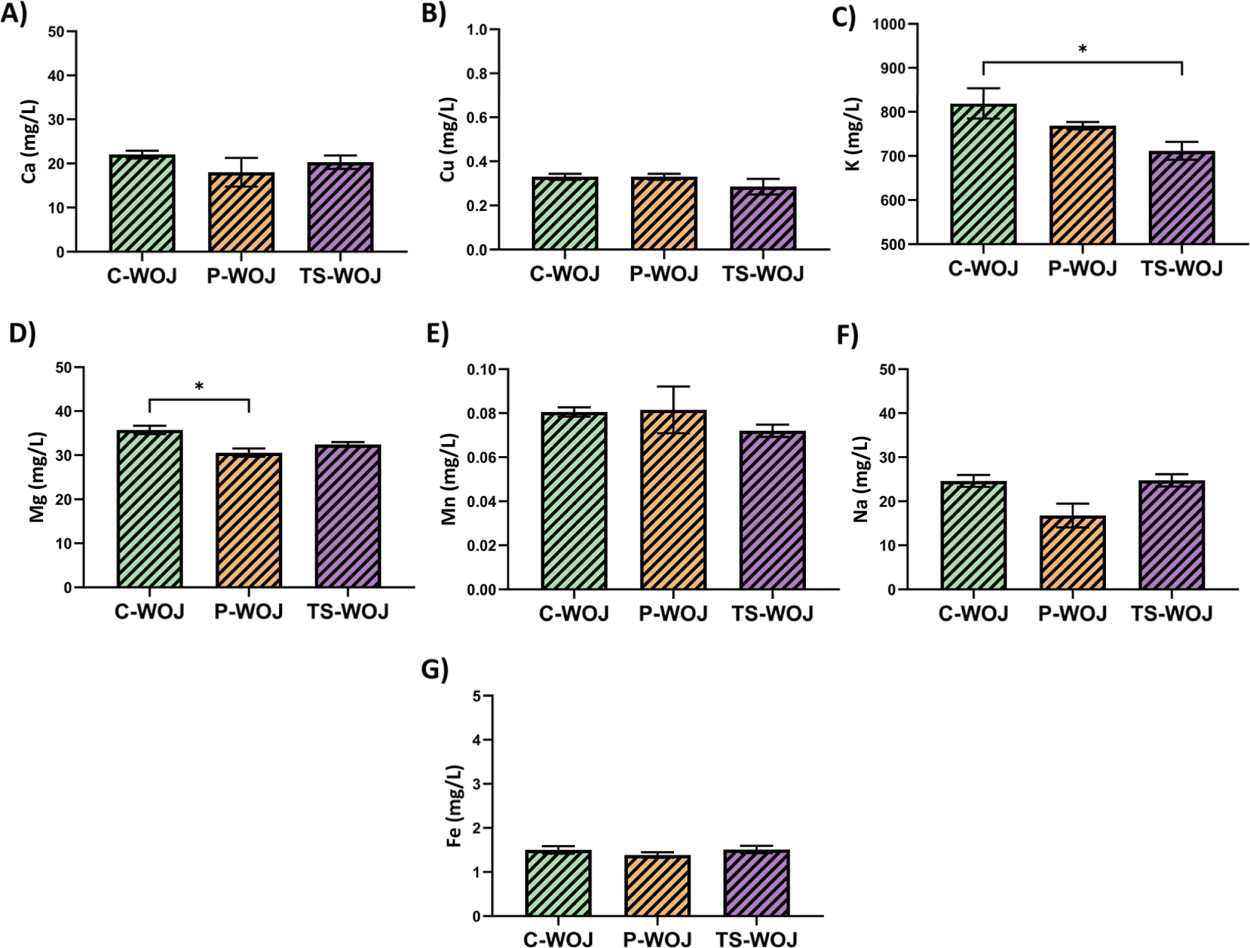
Results for Ca (A), Cu (B), K (C), Mg (D), Mn (E), Na (F), and Fe (G), minerals of C-WOJ, P-WOJ, and TS-WOJ *p < 0.05 (*n* = 3 ± SD). C-WOJ: control white onion juice; P-WOJ: thermal pasteurized white onion juice; TS-WOJ: thermosonication-treated white onion juice.

**Figure 4:**
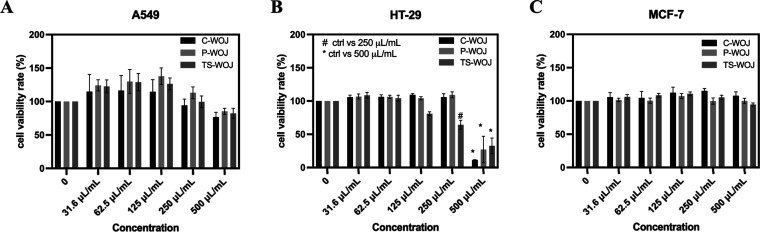
Effect of different concentrations of C-WOJ, P-WOJ, and TS-WOJ groups on cell viability in (A) A549 lung, (B) HT-29 colon and (C) MCF-7 breast cancer cells. Data was analyzed by one-way ANOVA (*n* = 3 ± SD). # Indicates ctrl (untreated control) vs 250 μL/mL concentration (*p* < 0.0001), * indicates ctrl (untreated control) vs 500 μL/mL concentration (*p* < 0.0001). C-WOJ: control white onion juice; P-WOJ: thermal pasteurized white onion juice; TS-WOJ: thermosonication-treated white onion juice.

Onion includes several phenolic and bioactive constituents, especially phenolics and flavonoids.^48^ The total flavonoid content (TFC) of white onion juices was measured, obtaining results of 16.33, 15.25, and 19.37 mg CE/100 mL for C-WOJ, P-WOJ, and TS-WOJ samples, respectively (Figure 4D). No significant variation was noted in the TFC contents of C-WOJ and P-WOJ samples; however, a difference was identified between P-WOJ and TS-WOJ samples (*p* < 0.05). Similar to our study, Das et al. (2023) reported a decrease in the TFC content of the pasteurized sample compared to the untreated control sample in their study with posotia leaf (V. negundo) juice^41^ With the application of ultrasound, cavitation (formation, increase and collapse of micro bubbles) occurs and the effect of temperature in the TS process (ultrasound combined with high temperatures) cause the bursting of bubbles in the treated product, leading to the formation and release of free radicals.^49^ Due to these ultrasonic waves, it is believed that the bioactive compounds in thermosonicated white onion juice samples may have increased.

### 3.3 Phenolic Compounds

Phenolic substances obtained from white onion juice samples, applied processes, and their values are shown in Table 4. In the study, chlorogenic acid, one of the phenolic compounds, was at the highest value in all samples. Chlorogenic acid value (TS-WOJ 355.71 ± 11.08 μg/mL) was found to be higher than the control (C-WOJ) and thermal pasteurization (P-WOJ) postapplication values in thermosonicated samples. Quercetin C-WOJ (181.42 ± 6.26) and P-WOJ (153.13 ± 7.32 μg/mL) were the second most abundant phenolic substances, and Catechine hydrate TS-WOJ (123.86 ± 5.92 μg/mL) was found to be the third most abundant. The thermosonication process positively affects the phenolic components chlorogenic acid, catechine hydrate, and *t*-cinamic acid values. Although the value of quercetin was high in the control sample, it was affected by the thermal pasteurization process and decreased considerably in quantity. In addition, thermosonication had less (negative) effects on quercetin than thermal pasteurization. In a study in which the total amount of phenolic substances was evaluated as a result of heating processes (80 °C, 120 °C, 150 °C; 30 min) applied to different onion varieties, it was stated that the number of phenolic substances increased up to 120 °C (according to the ambient temperature) and started to decrease at 150 °C. It is noted that the temperature treatment applied to the onions and different processes such as boiling, sautéing, frying, and baking cause the release of phenolic components and increased quantity. The formation of phenolic compounds, esterification, and glycosylation or Maillard reaction products 50 can explain this. In analyzing five different onion varieties, the phenolic substance was 4.75–5.32 mg GAE/g dw, and gallic acid (64.90 ± 0.1.22 mg/g dw) was determined as the highest phenolic substance.^50^ A study in white onion juice also stated the total phenolic content as 56–156 mg GAE/mL.^51^ In our study, 649.45 ± 25.13 μg/mL were found in TS-WOJ with the highest total phenolic substance value. The developed thermosonication technique enhances the overall quantity of phenolic compounds, particularly chlorogenic acid, catechin hydrate, and quercetin. Conversely, the overall concentration of phenolic compounds was decreased in the samples subjected to thermal pasteurization. In thermal pasteurization, it was observed that the concentrations of the phenolic compounds chlorogenic acid and catechin hydrate increased, whereas other phenolic substances, including quercetin (excluding caffeic acid), declined (Table 4).

**Table 4:** Organic acid, sugar, and phenolic compounds analysis results of C-WOJ, P-WOJ and TS-WOJ^a^

		samples		
studied compound		C-WOJ	P-WOJ	TS-WOJ
organic acid (μg/mL)	oxalic acid	0.04 ± 0.00	0.04 ± 0.00	0.05 ± 0.01
	citric acid	0.40 ± 0.04	0.40 ± 0.03	0.47 ± 0.01
	tartaric acid	0.22 ± 0.01^a^	0.30 ± 0.01^b^	0.43 ± 0.02^c^
	malic acid	6.22 ± 0.47	5.99 ± 0.35	6.09 ± 0.25
	succinic acid	1.80 ± 0.13^b^	1.31 ± 0.08^a^	1.57 ± 0.06^ab^
	fumaric acid	0.02 ± 0.00	0.04 ± 0.00	0.02 ± 0.00
	propionic acid	0.08 ± 0.01	0.06 ± 0.01	0.07 ± 0.00
	total	8.77 ± 0.65	8.12 ± 0.47	8.69 ± 0.36
sugars (μg/mL)	fructose	16.61 ± 0.18	16.35 ± 0.23	16.30 ± 0.30
	glucose	17.13 ± 0.27	16.24 ± 0.47	16.31 ± 0.21
	sucrose	2.03 ± 0.04^b^	1.60 ± 0.02^a^	2.00 ± 0.04^b^
	total	35.76 ± 0.47	34.18 ± 0.73	34.61 ± 0.54
phenolic compounds (μg/mL)	klorogenic acid	241.70 ± 8.34^a^	306.24 ± 6.09^b^	355.71 ± 11.08^c^
	catechine hydrate	95.47 ± 3.29^a^	104.63 ± 2.90^a^	123.86 ± 5.92^b^
	caffeic acid	0.00 ± 0.00	3.03 ± 0.08	0.00 ± 0.00
	*p*-coumaric acid	3.40 ± 0.12	0.00 ± 0.00	0.00 ± 0.00
	rutin	2.44 ± 0.10^b^	0.52 ± 0.01^a^	0.70 ± 0.03^a^
	*t*-ferulic acid	2.06 ± 0.08^b^	0.33 ± 0.01^a^	1.93 ± 0.09^b^
	naringin	46.02 ± 1.9^b^	0.95 ± 0.03^a^	0.35 ± 0.02^a^
	*o*-coumaric acid	15.49 ± 0.64^c^	7.77 ± 0.21^a^	10.54 ± 0.50b
	salicylic acid	4.21 ± 0.17	0.00 ± 0.00	0.00 ± 0.00
	resveratrol	0.45 ± 0.01^b^	0.02 ± 0.00^a^	0.48 ± 0.02^b^
	quercetin	181.42 ± 6.26^c^	77.97 ± 2.16^a^	^153.13 ± 7.32b^
	*t*-cinamic acid	0.75 ± 0.03^b^	0.57 ± 0.02^a^	1.06 ± 0.05^c^
	naringenin	0.08 ± 0.00^a^	0.10 ± 0.00^b^	0.00 ± 0.00
	4-hydroxy benzoic acid	0.42 ± 0.01^a^	0.00 ± 0.00	0.50 ± 0.02^b^
	rosmarinic acid	3.16 ± 0.11^b^	0.00 ± 0.00	0.92 ± 0.04^a^
	hydroxy sinamic acid	0.00 ± 0.00	0.00 ± 0.00	0.26 ± 0.01
**total**		**597.06** **±** **21.06** ^**b**^	**502.11** **±** **11.52** ^**a**^	**649.45 25.13** ^**b**^

a The results are the mean ± standard deviation (*n* = 3). The values marked with different letters within the line are significantly different from each other (*p* < 0.05). C-WOJ: control white onion juice; P-WOJ: thermal pasteurized white onion juice; TS-WOJ: thermosonication-treated white onion juice.

### 3.4 Organic Acid and Sugar Content

Table 4 presents the organic acid values measured in white onion juice, as well as those acquired following heat pasteurization and thermosonication treatments. Malic acid was identified as the predominant organic acid in white onion. In comparison to heat pasteurization and thermosonication, C-WOJ exhibited a superior value of 6.22 ± 0.47 μg/mL in control samples. Succinic acid values were 1.80 ± 0.13, 1.31 ± 0.08, and 1.57 ± 0.06 μg/mL, respectively. Total organic acids were highest in the control sample and in the samples that were subsequently thermosonicated. Malic acid is the predominant organic acid in onions, according to a study.^50^ In the study in which the organic acid amounts of onion samples obtained from different regions were examined,^52^ significant differences in organic acids were observed among onion types. Their results stated that citric acid is 48.5 ± 241 mg/100 g, and malic acid is 43.6 ± 10.4 mg/100 g.

It is stated that carbohydrates make up a significant proportion (9.3%) of dry matter in onions.^51^ The sugars and amounts determined in onion juice after thermal pasteurization and thermosonication applications are shown in Table 4. Glucose was higher than other sugars in onion juice samples examined for essential carbohydrates for fructose, glucose, and sucrose. The highest glucose value was 17.13 ± 0.27 μg/mL in the control group (C-WOJ). The control group showed the highest value in total sugar amounts. The process of thermal pasteurization and thermosonication has resulted in a decrease in the amount of sugars. The thermosonication method affected the values less than thermal pasteurization (except fructose). In their study, Sharma et al. 2015 evaluated glucose as the highest sugar despite heat treatment, and in the 2007 study of Liguori et al., they evaluated fructose as the most abundant sugar in onions. Our study assessed that the reduction in sugar content during thermal pasteurization may be attributed to the heating effect and the Maillard process.^50,53^ A study stated that the total sugar in white onions was 513.9 g/kg DM in the average dry matter.^54^ In the study, which used eight different types of onions, the total amount of sugar was between 2.62% and 4.72%.^55^ In our study, 35.76 μg/mL was detected in the total glucose control sample. The sugar content was affected by the processes applied.

### 3.5 Analysis of Minerals

Mineral findings obtained from C-WOJ, P-WOJ, and TS-WOJ groups are shown in Figure 3. In all sample groups, C-WOJ (819.09 mg/L), P-WOJ (768.56 mg/L), and TS-WOJ (711.50 mg/L), the mineral obtained at the highest level is Potassium (K). In all sample groups, C-WOJ (0.08 mg/L), P-WOJ (0.08 mg/L), and TS-WOJ (0.07 mg/L), the mineral obtained at the lowest level is Manganese (Mn). From C-WOJ, P-WOJ, TS-WOJ groups, respectively (mg/L) Ca (22.01; 18.03; 20.30); Cu (0.33; 0.33; 0.28); K (819.09; 768.56; 711.50); Mg (35.72; 30.58; 32.44); Mn (0.08; 0.08; 0.07); Na (24.62; 16.76; 24.75); Fe (1.50; 1.38; 1.51) mineral substances were obtained. As a result of the applied thermal and nonthermal applications, a statistically significant change was observed only for K mineral between C-WOJ and TS-WOJ groups and for Mg mineral between C-WOJ and P-WOJ groups (*p* < 0.05). Various studies suggest that the most abundant minerals found in onions; Potassium,^54,56–58^ Calcium,^59^ Sodium^60^ and these findings are generally consistent with our results. It is thought that the amount and type of minerals vary depending on the growing conditions and geographical areas. Potassium is an essential element required for maintaining acid and electrolyte balance, cell functions and total body fluid volume. According to,^61^ adults’ recommended daily potassium intake is 3510 mg. Accordingly, onion can be considered a good food source in terms of potassium. The data shows that onion is a mineral-rich food for healthy nutrition and can be preferred in daily nutrition and gastronomy. Since nonthermal processes generally reduce mineral losses, it is predicted that they are suitable for preference in the food sector.

### 3.6 Analysis of Amino Acids

The amino acid levels in white onion juice for the C-WOJ, P-WOJ, and TS-WOJ groups are presented in Table 5. All groups exhibited varying quantities of the amino acids Alanine, Arginine, Aspartic acid, Glutamic acid, Histidine, Isoleucine, Leucine, Methionine, Ornithine, Phenylalanine, Proline, Serine, Threonine, Tyrosine, and Valine. Cystine and Taurine amino acids were absent in several sample groups, however Lysine amino acid was only undetected in the P-WOJ group. The total amino acid levels were quantified as follows: C-WOJ (17.80 ± 0.09 mg/100 g), TS-WOJ (15.64 ± 0.68 mg/100 g), and P-WOJ (15.42 ± 0.29 mg/100 g), arranged from highest to lowest. In comparison to the C-WOJ group, the P-WOJ group exhibited a reduction in total amino acid levels of 2.38 mg/100 g, while the TS-WOJ group demonstrated a decrease of 2.16 mg/100 g. Since thermosonication is a nonthermal process, amino acid loss was lower than with pasteurization. The higher total amino acid loss in the P-WOJ group is attributed to the decomposition of amino acids due to pasteurization. Aspartic Acid was the amino acid identified at the greatest concentration across all sample groups. Methionine was identified as the amino acid present in the lowest concentration in the C-WOJ and TS-WOJ groups. Arginine was identified as the amino acid present at the lowest concentration in the P-WOJ group. In contrast to the C-WOJ group, the other two groups exhibited statistically significant alterations in amino acid levels (*p* < 0.05). Upon examination of the sample groups, it was determined that they included essential amino acids such as Histidine, Isoleucine, Leucine, Lysine (excluding the P-WOJ group), Methionine, Phenylalanine, Threonine, and Valine, in addition to nonessential amino acids (Alanine, Arginine, Aspartic Acid, Cystine, Glutamic Acid, Glycine, Ornithine, Proline, Serine, Tyrosine, Taurine). While Okonkwo et al., 2022 ^62^ detected the highest levels of Leucine (4.11 g/100 g) and Arginine (3.74 g/100 g) amino acids in onion extracts;^63^ detected the highest levels of Arginine (16.492 ± 0.256 mg/g DW) and Glutamic acid (14.964 ± 0.008 mg/g DW) amino acids in their study on hybrid onions. Although the amino acid profiles obtained as a result of our research show similarities, it is thought that the differences observed in quantity are due to the plant’s cultivation methods and geographical structure. In addition to their nutritional properties, amino acids contribute to the umami taste. Glutamic acid is one of the amino acids most effective in eliciting the umami taste.^64^ According to our research findings, the amino acid found at the second highest level in all analysis groups is glutamic acid, which confirms the findings of other researchers.

**Table 5:** Amino Acid Content Analysis Results of C-WOJ, P-WOJ and TS-WOJ^a^

no	amino acid content (mg/100 g)	C-WOJ	P-WOJ	TS-WOJ
1	alanine	1.45 ± 0.04^b^	0.95 ± 0.09^a^	1.13 ± 0.09^ab^
2	arginine	0.06 ± 0.01^b^	0.01 ± 0.00^a^	0.06 ± 0.01^b^
3	aspartic acid	3.87 ± 0.18^a^	3.28 ± 0.21^a^	3.82 ± 0.41^a^
4	cystine	0.00 ± 0.00	0.00 ± 0.00	0.00 ± 0.00
5	glutamic acid	3.27 ± 0.03^c^	2.93 ± 0.03^b^	2.56 ± 0.11^a^
6	glycine	0.00 ± 0.00	0.00 ± 0.00	0.00 ± 0.00
7	histidine	0.44 ± 0.01^a^	0.50 ± 0.00^b^	0.45 ± 0.01^a^
8	isoleucine	0.04 ± 0.01^a^	0.11 ± 0.01^b^	0.07 ± 0.01^a^
9	leucine	0.21 ± 0.03^a^	0.49 ± 0.01^b^	0.21 ± 0.01^a^
10	lysine	0.12 ± 0.02^b^	0.00 ± 0.00^a^	0.22 ± 0.01^c^
11	methionine	0.03 ± 0.00^a^	0.03 ± 0.01^a^	0.03 ± 0.00^a^
12	ornitine	1.09 ± 0.01^b^	1.00 ± 0.04^b^	0.31 ± 0.01^a^
13	phenylalanine	0.39 ± 0.00^b^	0.62 ± 0.01^c^	0.31 ± 0.01^a^
14	proline	2.02 ± 0.02^b^	1.45 ± 0.01^a^	2.15 ± 0.01^c^
15	serine	2.07 ± 0.07^b^	1.72 ± 0.08^a^	1.70 ± 0.04^a^
16	threonine	2.08 ± 0.08^b^	1.6 ± 0.02^a^	1.96 ± 0.01^b^
17	tyrosine	0.45 ± 0.01^b^	0.45 ± 0.01^b^	0.40 ± 0.00^a^
18	valine	0.24 ± 0.01^a^	0.30 ± 0.00^b^	0.30 ± 0.01^b^
19	taurine	0.00 ± 0.00	0.00 ± 0.00	0.00 ± 0.00
total		17.80 ± 0.09^b^	15.42 ± 0.29^a^	15.64 ± 0.68^a^

a The results are the mean ± standard deviation (*n* = 3). The values marked with different letters within the line are significantly different from each other (*p* < 0.05). C-WOJ: control white onion juice; P-WOJ: thermal pasteurized white onion juice; TS-WOJ: thermosonication-treated white onion juice.

### 3.7 Polyphenol Oxidase Enzyme (PPO) Activity

In the study, it was found that the percentage of white onion juice polyphenol oxidase (PPO) activity decreased in the P-WOJ group (40.06%) compared to the C-WOJ (73.00%) group and this decrease was statistically significant (*p* < 0.001). Similarly, a statistically significant decrease was observed in the C-WOJ (73.00%) polyphenol oxidase activation in the TS-WOJ (43.07%) group (*p* < 0.001). No significant difference was seen between the TS-WOJ and P-WOJ groups concerning the percentage of polyphenol oxidase activity. In the study, it can be said that both thermal pasteurization and thermosonication processes are effective in PPO inactivation. Anaya-Esparza et al. (2017)^9^ investigated the processing of graviola nectar using thermosonication, assessing the inactivation of PPO and its impact on quality measures. They determined that thermosonication could serve as an exceptional alternative, maintaining both total PPO inactivation and the quality of graviola fruit nectar. Baltacioglu (2021)^65^ indicated in their research on the impact of thermosonication on peach juice that inactivation of the PPO enzyme occurred at lower temperatures with thermosonication than with thermal processing.

Another investigation assessed the synergistic impact of ultrasound, mild heat, and high pressure on Escherichia coli O157:H7, polyphenol oxidase (PPO), and anthocyanin levels in fresh blueberry juice, revealing that manothermosonication enhanced the inactivation of the PPO enzyme when combined with high pressure and mild heat.^66^

### 3.8 Antihypertensive (ACE-Inhibitor), and Antidiabetic Activity

Diabetes problems are implicated in the cause of cardiovascular illnesses.^67,68^ The treatment of hypertension involves the inhibition of the Angiotensin-converting enzyme (ACE), which obstructs the conversion of angiotensin I to angiotensin II. The therapy strategy for type 2 diabetes involves decelerating digestion and absorption by blocking α-amylase and α-glucosidase, enzymes responsible for carbohydrate breakdown.^69^ In the study, in the evaluation of ACE inhibition activity of white onion juice, a statistically significant decrease was observed in the P-WOJ (21.65%) group compared to the TS-WOJ (25.03%) group (*p* < 0.001).

The ACE inhibition activity exhibited a substantial increase in the TS-WOJ group (25.03%) relative to the C-WOJ group (22.82%) (*p* < 0.05). The reduction noted in the P-WOJ (21.65%) group relative to the C-WOJ (22.82%) group was deemed statistically insignificant. In the study, it can be said that the thermosonication process gave better results in ACE inhibition activity than the thermal pasteurization process. Yikmis et al. (2024) detected an enhancement in antihypertensive and antidiabetic properties following the thermal pasteurization and thermosonication of purple onion juice in their investigation of the anticancer, antibacterial, ascorbic acid, antihypertensive, and antidiabetic effects of thermosonicated and thermal pasteurized purple onion juice.^15^

The investigation revealed a statistically significant reduction in α-glucosidase inhibitory activity in the P-WOJ (37.95%) group compared to the TS-WOJ (43.11%) group (*p* < 0.01). The TS-WOJ group exhibited a notable increase in α-glucosidase inhibitory activity, achieving 43.11%, in contrast to the C-WOJ group, which measured 40.45% (*p* < 0.05). The reduction in the P-WOJ group (37.95%) was not statistically significant when compared to the C-WOJ group (40.45%). In terms of α**-**amylase inhibitory activity in white onion juice, a statistically significant decrease was observed in the P-WOJ (32.80%) group compared to the TS-WOJ (36.21%) group (*p* < 0.01). A significant increase in α-amylase inhibitory activity was detected in the TS-WOJ group (36.21%) compared to the C-WOJ (34.33%) group (*p* < 0.05). The decrease observed in the P-WOJ (32.80%) group was not statistically significant compared to the C-WOJ (34.33%) group. Oboh et al. (2018) highlighted that white onion exhibits a greater inhibitory effect on α-amylase activity compared to purple onion and garlic in their research on the management mechanisms of diabetes and hypertension.^69^ Scudino et al. (2023) compared thermosonication and traditional pasteurization applied to raw milk to produce Minas Frescal cheese in a study they conducted. The study results matched with our findings, indicating that thermosonication exhibited more inhibitory action against α-glucosidase and α-amylase enzymes.^70^

### 3.9 Anticancer Activity

MTT assay was first performed to assess the cytotoxic effects of WOJ samples on the cancer cells. Subsequently, wound healing, colony formation, apoptosis, and DNA fragmentation assays were performed. In A549 lung cancer cells, it was observed that a 500 μL/mL concentration induced cell death in all groups; however, this effect was not statistically significant (Figure 4A). In HT-29 colon cancer cells, it was shown that 500 μL/mL concentration induced cell death in all three groups (4B). In addition, it was determined that lower concentration (250 μL/mL) induced cell death in the TS-WOJ group. The effect of C-WOJ, P-WOJ, and TS-WOJ groups on cell viability in MCF7 breast cancer cells has also been determined, but it was found that none of the three groups affected MCF-7 cells (Figure 4C). Due to the ineffectiveness of samples in breast cancer cells, no further analyses were conducted.

The impact of C-WOJ, P-WOJ, and TS-WOJ samples on apoptosis in A549 cells was assessed using Annexin V/PI double labeling and analyzed via flow cytometry. The apoptotic cell percentages were 10.6% in the control group and 12.3%, 10.1%, and 14.5% in the C-WOJ, P-WOJ, and TS-WOJ groups, respectively (Figure 5A). On the other hand, colony formation assays were conducted in A549 cells, and it was found that the C-WOJ, P-WOJ, and TS-WOJ inhibited colony formation (*p* < 0.0001) (Figure 5B). Moreover, the effects of C-WOJ, P-WOJ, and TS-WOJ on DNA fragmentation were investigated to reveal further the apoptotic effect of WOJ samples (Figure 5C). The impact of WOJ samples on wound healing of A549 cells was shown in Figure 5D; the wound gap was measured and analyzed in triplicate using the ImageJ software. The wound gap in the control (untreated) group was fully closed by day four, whereas the groups exposed to C-WOJ, P-WOJ, and TS-WOJ exhibited incomplete wound closure (Supplementary Figure S1). Additionally, although a substantial disparity existed between the C-WOJ, P-WOJ, and TS-WOJ groups and the control group, no significant difference was observed across the various WOJ samples. The results from A549 cells indicated that WOJ inhibited cancer cell growth by inducing cell death; however, no significant differences were observed among the C-WOJ, P-WOJ, and TS-WOJ groups.

**Figure 5:**
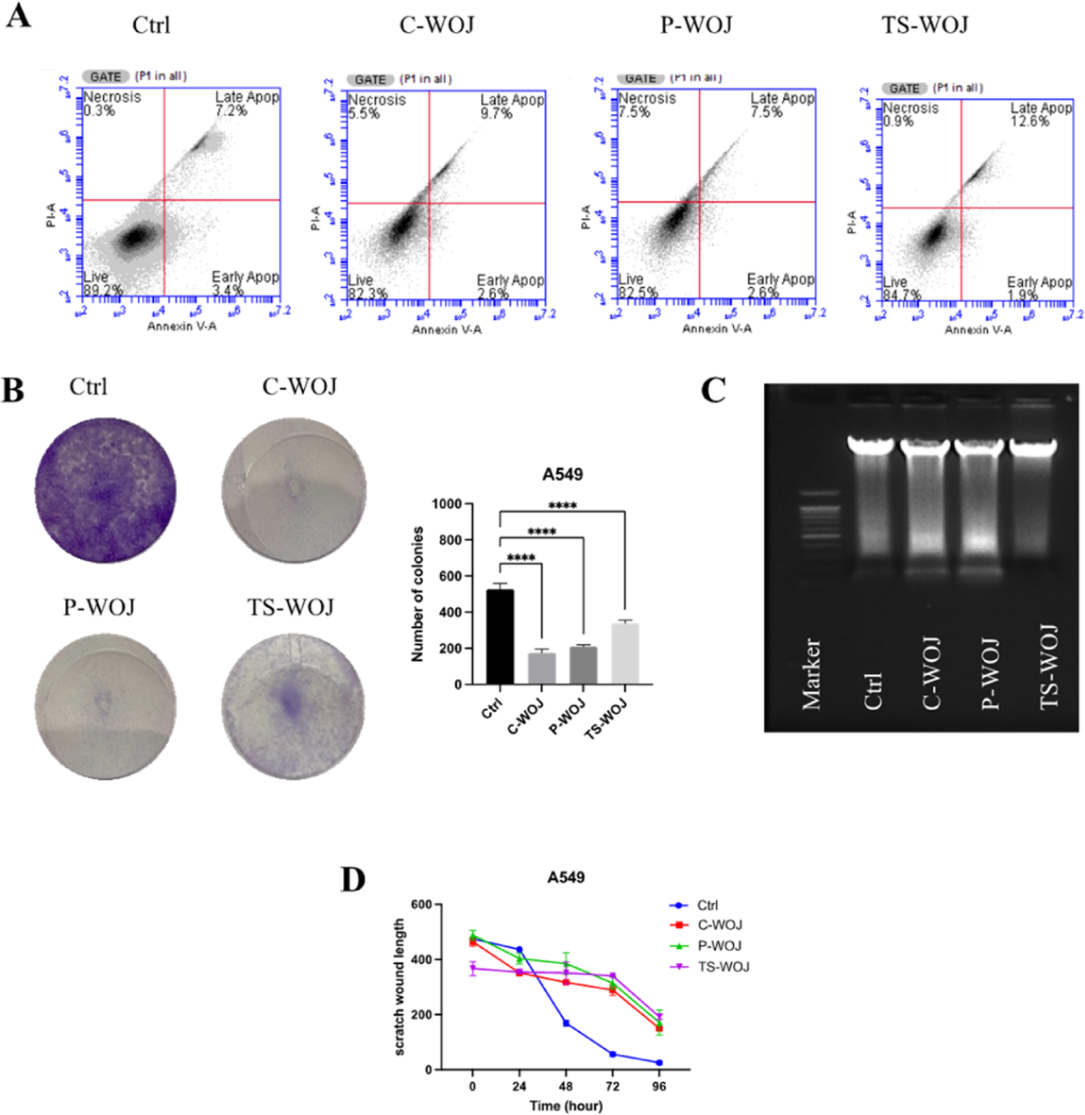
Effect of different C-WOJ, P-WOJ and TS-WOJ on (A) apoptotic cell death, (B) colony formation, (C) DNA fragmentation and (D) wound closure in A549 cells. For apoptosis, DNA fragmentation and wound closure assay experiments, a concentration of 250 μL/mL was used, and a concentration of 50 μL/mL was used for colony formation experiments. **** indicates *p* < 0.0001. Ctrl: untreated control; C-WOJ: control white onion juice; P-WOJ: thermal pasteurized white onion juice; TS-WOJ: thermosonication-treated white onion juice.

According to the MTT findings, WOJ was most effective in HT-29 cells. Next, the effects of C-WOJ, P-WOJ, and TS-WOJ onion juice samples on apoptosis in HT-29 cells have been determined. It was determined that the apoptotic cell ratio was 3.0% in the control group, 7.6% in the C-WOJ group, 7.1% in the P-WOJ group, and 11.9% in the TS-WOJ group (Figure 6A). According to the colony formation assay results of HT-29 cells, colony formation was suppressed compared to the control group (Figure 6B). In colony formation experiments, immature colonies were higher in C-WOJ and P-WOJ groups than in the others. This can be explained as the reason why TS-WOJ had more colonies compared to C-WOJ and P-WOJ groups in colony formation experiments.

**Figure 6:**
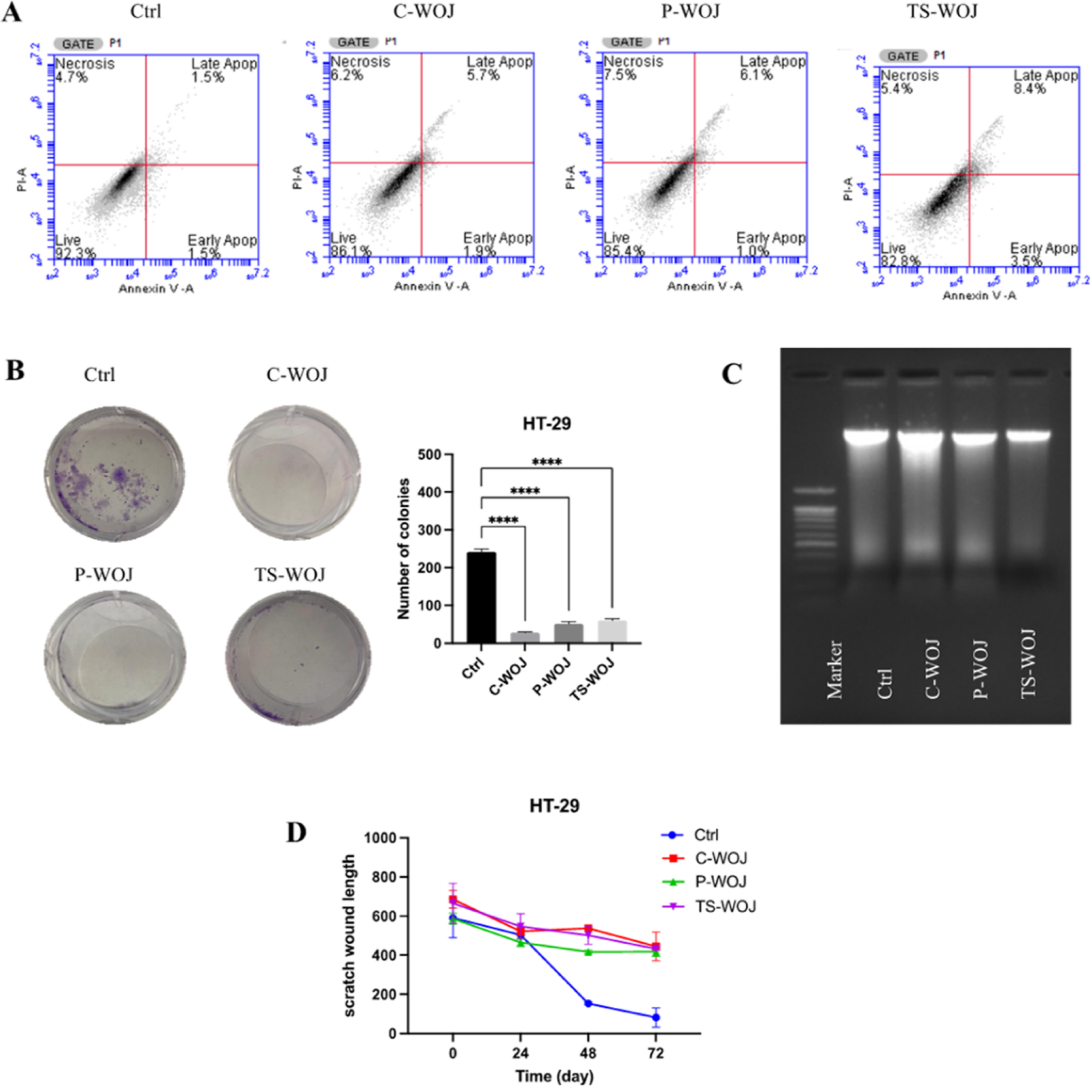
Effect of different C-WOJ, P-WOJ and TS-WOJ on (A) apoptotic cell death, (B) colony formation, (C) DNA fragmentation and (D) wound closure in Ht-29 colon cancer cells. Apoptosis, DNA fragmentation, and wound closure assay experiments were conducted at a concentration of 250 μL/mL, while colony formation assays were performed at a concentration of 50 μL/mL. **** indicates *p* < 0.0001. Ctrl: untreated control; C-WOJ: control white onion juice; P-WOJ: thermal pasteurized white onion juice; TS-WOJ: thermosonication-treated white onion.

In HT-29 wound healing experiments, the wounds created in the control group closed after 72 h, while complete closure was not observed in the groups exposed to C-WOJ, P-WOJ and TS-WOJ. When the groups were evaluated within themselves, it was observed that the gap was higher in the TS-WOJ group at the end of 72 h (Figure 6D and Supplementary Figure S2). When all the findings were evaluated together in HT-29 cells, it was found that TS-WOJ samples have a higher anticancer effect on colon cancer cells than the others.

Some of the figures (effect of different onion juice samples on the wound healing process in A549 cells; effect of different onion juice samples on the wound healing process in HT-29 cells) used in this study are detailed in the supplementary file, specifically in Figures S1 and S2.

Onions possess a distinctive assortment of phytochemicals, including beneficial sulfur-containing substances like sulfonic acids and thiosulfinates, alongside phenolic acids and flavonoids such as quercetin and kaempferol. It also comprises certain saccharides, proteins, and vitamins including B1, B2, and C, in addition to potassium and selenium. Epidemiological studies indicate a correlation between enhanced onion consumption and decreased cancer risk; nevertheless, these findings are constrained. However, studies have shown that onion consumption protects against digestive system cancers.^71^ Edible wild onions demonstrate significant potential by exhibiting a synergistic anticancer effect with doxorubicin in human hepatoma (HepG2) and lung carcinoma (A549) cells. This plant has shown efficacy in preserving against doxorubicin-induced cytotoxicity in human normal fibroblast cells (MRC-5) and in vivo zebrafish models.^72^ Furthermore, in breast cancer patients administered doxorubicin, it has been documented that the intake of fresh onions decreased fasting blood glucose levels and enhanced insulin sensitivity.^73^ Our previous study examined the anticancer activity of purple onion juice in A549, HeLa, and Caco-2 cells. The results suggested that, in comparison to the control group, it had no significant effect on cell mortality.^15^ Furthermore, another study found that purple onion vinegar, when applied to HGC-27 gastric cancer cells and HCT-116 colon cancer cells using different preparation methods, had no significant impact on cell viability.^30^ This study investigated the anticancer effects of white onion juice samples obtained through different techniques on A549 lung cancer, HT-29 colon cancer, and MCF-7 breast cancer cell lines. The findings indicate that WOJ samples have varying effectiveness on different cancer cell types. Among the cancer cells studied, the most significant result was observed in the colon cancer cells. In light of these findings, further detailed investigation of the anticancer properties of WOJ is necessary across different cancer types, supported by in vivo animal models. Finally, it is suggested that WOJ samples be considered not as a primary treatment but an adjunctive therapy in colon cancer treatment.

## 4 Conclusions

Ultrasound technology is an alternative to heat treatment that offers a sustainable option with low energy consumption, making it an environmentally friendly method. Ultrasound, as an emerging technology, exhibits exceptional capabilities in diminishing harmful microorganisms in vegetable juices, enhancing food safety, prolonging product shelf life, inhibiting undesirable enzyme activity, and maintaining the color, aroma, flavor, and nutritional attributes of the product.

Thermosonication technology is an innovative method that works by the combined use of ultrasound waves and heating. This research demonstrates that the thermosonication (TS) technique markedly improves the bioactive properties and functional constituents of white onion (A. cepa L.) juice. Our research demonstrates that thermosonication significantly enhances total phenolic content (TPC), total flavonoid content (TFC), and antioxidant activity (DPPH) under optimized conditions (45.45 °C, 12.90 min, 67.67% amplitude). The TS process increased bioactive compounds of white onion juice, notably chlorogenic acid (355.71 μg/mL), catechin hydrate (123.86 μg/mL), and quercetin (153.13 μg/mL), while also improving anticancer, antihypertensive, and antidiabetic effects. Furthermore, there was a substantial enhancement in the overall amino acid content, particularly in the amounts of aspartic acid and glutamic acid. The results indicates that onion is a mineral-dense food beneficial for optimal nutrition and can be favored in everyday dietary practices and culinary applications. Nonthermal procedures typically minimize mineral losses, suggesting their preferential suitability in the food industry.

The TS procedure markedly enhanced anticancer efficacy by augmenting apoptotic cell death in colon cancer cells. Thermosonicated white onion juice demonstrated significant anticancer (inducing 11.9% apoptotic cell death in colon cancer cells). The antihypertensive (ACE-inhibitor) and antidiabetic (α-glucosidase and α-amylase inhibitor) activity were elevated in samples treated with TS. The findings demonstrate that thermosonication is an efficacious technique for enhancing the bioactive constituents and functional attributes of white onion juice, presenting substantial potential for the creation of functional products within the food industry.

Thermosonication technology to be used in innovative applications in vegetable and fruit juices in the future has the potential to increase quality and consumer satisfaction in the food sector. Thermosonication technology will be increasingly favored in technical and scientific domains for enhancing nutritional quality through superior preservation of vitamins, minerals, and antioxidants in food; formulating functional beverages; activating enzymes; generating novel taste and texture profiles; prolonging shelf life; and promoting sustainable production. Future research should thoroughly examine the effects of thermosonication on various onion varieties and other vegetable juices, as well as assess the molecular pathways behind their anticancer and antidiabetic properties utilizing in vivo models.

## Supplementary Material



## Notes

The some Figures used in this study are detailed in the Supporting Information file (Supplementary Figures S1 and S2); effect of different onion juice samples on the wound healing process in A549 cells; effect of different onion juice samples on the wound healing process in HT-29 cells (PDF)
