# Supplementary material for: Thermosonication as a Novel Processing Technique to Enhance Phenolic Content, Amino Acids, and Health-Promoting Activities of White Onion Juice
Source: ACS Omega. 2025 Jun 3;10(23):25051–67. doi: 10.1021/acsomega.5c03006 (PMC12177601; doi:10.1021/acsomega.5c03006)
Supplement: Supplementary file 1 [file ao5c03006_si_001.pdf]

Supporting Information

Thermosonication as a Novel Processing Technique to Enhance Phenolic Content, Amino Acids, and Health-Promoting Activities of White Onion Juice

Dilek DÜLGER ALTINER[1*], Seydi YIKMIŞ[2*], Esra BOZGEYİK[3], Melikenur TÜRKOL[4*], Filiz AKSU[5], Sema SANDIKÇI ALTUNATMAZ[6], Deniz AKTARAN BALA[7], Selim ÖĞÜT[8]

[1] *Department of Gastronomy and Culinary Arts, Tourism Faculty, Kocaeli University, 41400, Kartepe, Kocaeli, Türkiye*

[2] *Department of Food Technology, Tekirdag Namik Kemal University, Tekirdag, 59830, Türkiye*

[3] *Department of Medical Biology, Faculty of Medicine, Adiyaman University, 02200, Adiyaman, Türkiye*

[4] *Department of Nutrition and Dietetics, Faculty of Health Sciences, Tekirdag Namık Kemal University 59030 Tekirdag, Türkiye*

[5,6,7] *Department of Food Processing, Vocational School of Veterinary Medicine, Istanbul University-Cerrahpaşa, Avcilar, Istanbul, Türkiye*

[8] *Department of Biophysics, Faculty of Medicine, Bandırma Onyedi Eylul University, 10250, Bandırma, Balıkesir*

∗Corresponding Authors: Dilek Dülger Altıner (dilek.dulger@kocaeli.edu.tr) ORCID ID: 0000-0002-7043-2883; Seydi Yıkmış (syikmis@nku.edu.tr); Melikenur Türkol (melikenurturkol@gmail.com)

Contents

1.Figure S1. Effect of different onion juice samples on the wound healing process in A549 cells……………………………………………………………………………………….S3

2.Figure S2. Effect of different onion juice samples on the wound healing process in HT-29 cells……………………………………………………………………………………….S4

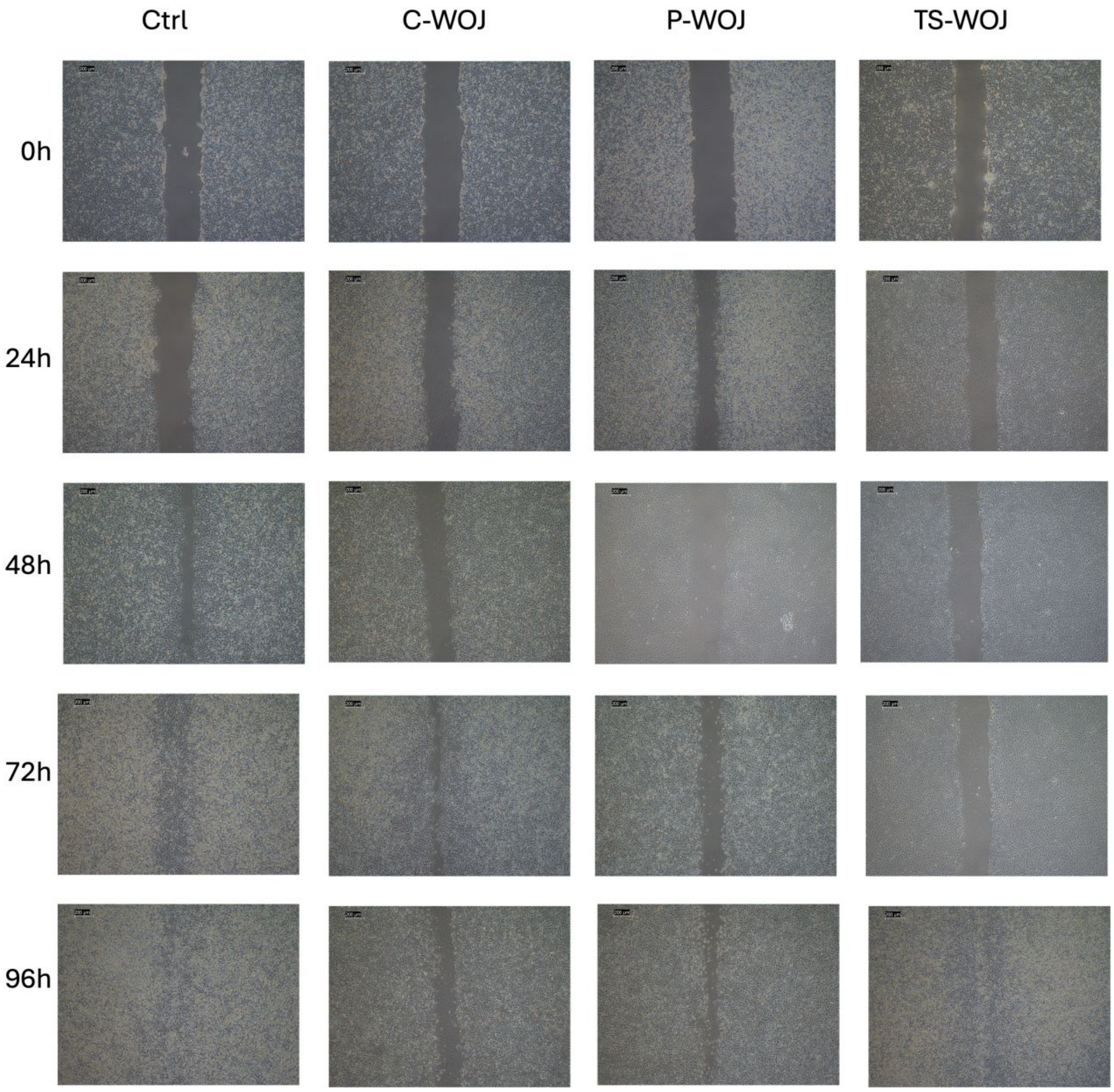

Figure S1. Effect of different onion juice samples on the wound healing process in A549 cells

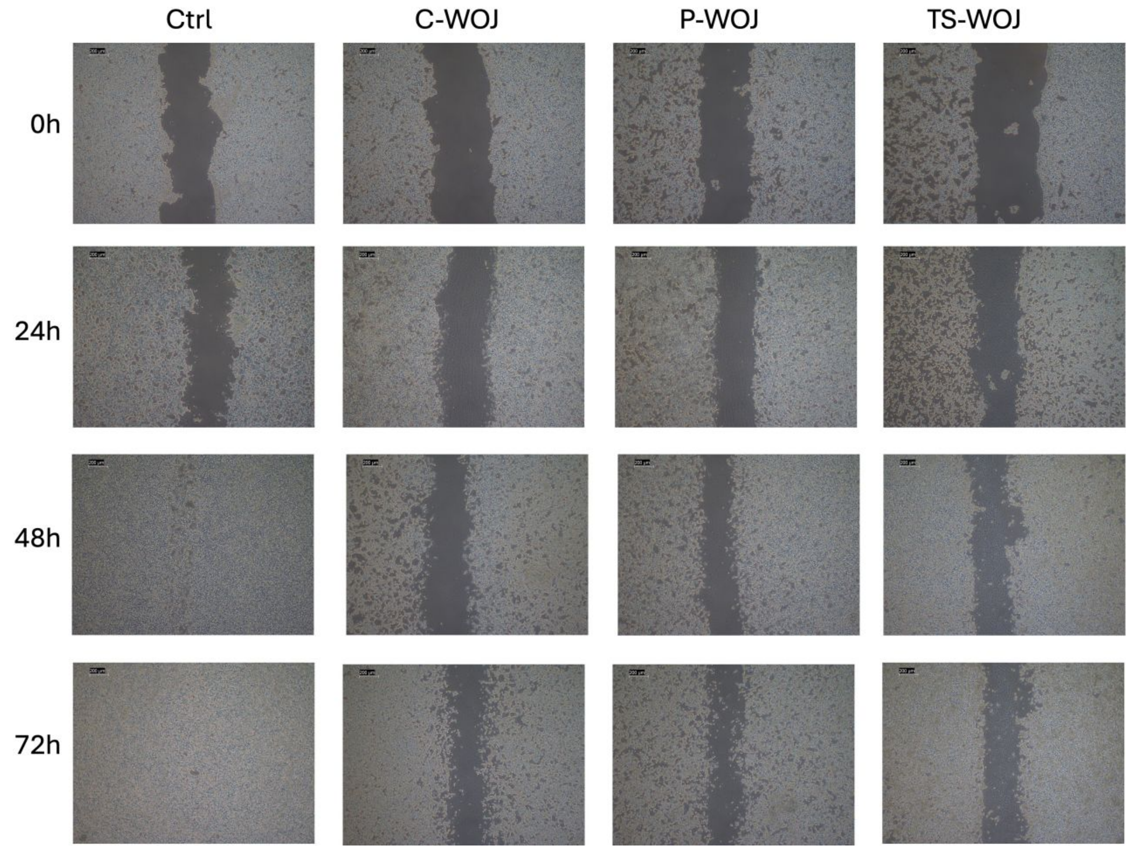

Figure S2. Effect of different onion juice samples on the wound healing process in HT-29 cells
